# Device simulations with A U-Net model predicting physical quantities in two-dimensional landscapes

**DOI:** 10.1038/s41598-023-27599-z

**Published:** 2023-01-13

**Authors:** Wen-Jay Lee, Wu-Tsung Hsieh, Bin-Horn Fang, Kuo-Hsing Kao, Nan-Yow Chen

**Affiliations:** 1 National Center for High-performance Computing, Hsinchu, Taiwan (R.O.C.); 2 Department of Electrical Engineering, National Cheng Kung University, Tainan, Taiwan (R.O.C.)

**Keywords:** Electrical and electronic engineering, Computational science

## Abstract

Although Technology Computer-Aided Design (TCAD) simulation has paved a successful and efficient way to significantly reduce the cost of experiments under the device design, it still encounters many challenges as the semiconductor industry goes through rapid development in recent years, i.e. Complex 3D device structures, power devices. Recently, although machine learning has been proposed to enable the simulation acceleration and inverse‑design of devices, which can quickly and accurately predict device performance, up to now physical quantities (such as electric field, potential energy, quantum-mechanically confined carrier distributions, and so on) being essential for understanding device physics can still only be obtained by traditional time-consuming self-consistent calculation. In this work, we employ a modified U-Net and train the models to predict the physical quantities of a MOSFET in two-dimensional landscapes for the first time. Errors in predictions by the two models have been analyzed, which shows the importance of a sufficient amount of data to prediction accuracy. The computation time for one landscape prediction with high accuracy by our well-trained U-Net model is much faster than the traditional approach. This work paves the way for interpretable predictions of device simulations based on convolutional neural networks.

## Introduction

Since the new Integrated Circuit technologies keep scaling down, the design of the new MOSFET device becomes more challenging. Technology Computer-Aided Design (TCAD), as an electronic design automation technique, using the finite element method to consistently solve the Poisson and Schrödinger equations provides an easy-access way to simulate the process and device of semiconductors. This method paved a successful way to avoid the numerous experiments for optimization and to dramatically reduce the cost from trial-and-error. In addition, it can determine the detailed electrical characteristics for understanding the device’s physics through simulation. However, with the rapid development of chip, the simulation of complex device such as three-dimensional (3D) device become more and more time consuming. The optimization of the device design through a production run becomes less possible.

Recently, neural-network based (NN-based) models being able to significantly shorten the device research and development process have attracted much attention because of their fast and accurate predictions for device electrical characteristics, statistical variability, sensitivity analysis, optimization, and reverse engineering^1–13^. These predictions rely on convolutional NN (CNN) or deep neural network (DNN). Normally, the DNN is not able to provide spatial relationships between data points due to the fact that each point in one layer is connected to each point in the next layer in the network locally. As a consequence, there is no direct access to device physics from these NN models for in-depth physical insights into device operation, which can be revealed by these physical quantities. Han et al.^14^ used CNN-based generative adversarial nets (DCGAN) to generate an initial guess of approximate electrostatic potential of device for avoiding huge computation and achieving a rapid convergence of simulation model of 2-D MOSFET. Using the electric potential as the initial solution for simulations, continuity equations can be solved to calculate the electron and hole densities under the fixed electrostatic potential profile in TCAD efficiently. However, we point out that the generator uses the device characteristics as the inputs to decode the output of the electrostatic potential profiles. There seems no complete structural and physical correlation between input and output. Despite the deep learning has been widely used in device design, there is no report demonstrating a method to predict the spatial physical quantities in a multidimensional landscape, e.g. spatial distributions of the electric field, potential energy, charge carrier density and so on in a device.

Towards interpretable device simulations, in addition to electrical characteristics, some crucial physical quantities of devices, such as electric field and potential energy, must be accurately predicted as well. This is because these quantities can be used to interpret short channel effects and currents induced by inter-band transitions^15^. Charge carrier distribution may be quantum-mechanically associated with charge scattering and gate controllability^16^. Hence, this work aims to realize accurate predictions of two-dimensional (2D) landscapes for these essential physical quantities through a CNN-based model^17^.

## Methodology

There are two main steps when developing a deep learning model for device simulations, which in sequence are training data generation and model training. The training data may be collected systematically by self-consistent simulations^1–11^ or measurements^12,13^. In this work, we choose the former approach based on a TCAD simulator^18^ to easily access the physical quantities. For the model training, we employ a U-Net, which is derived from the CNN and has been widely used in image segmentation^19^. These two steps of this work are detailed below.

**Figure 1:**
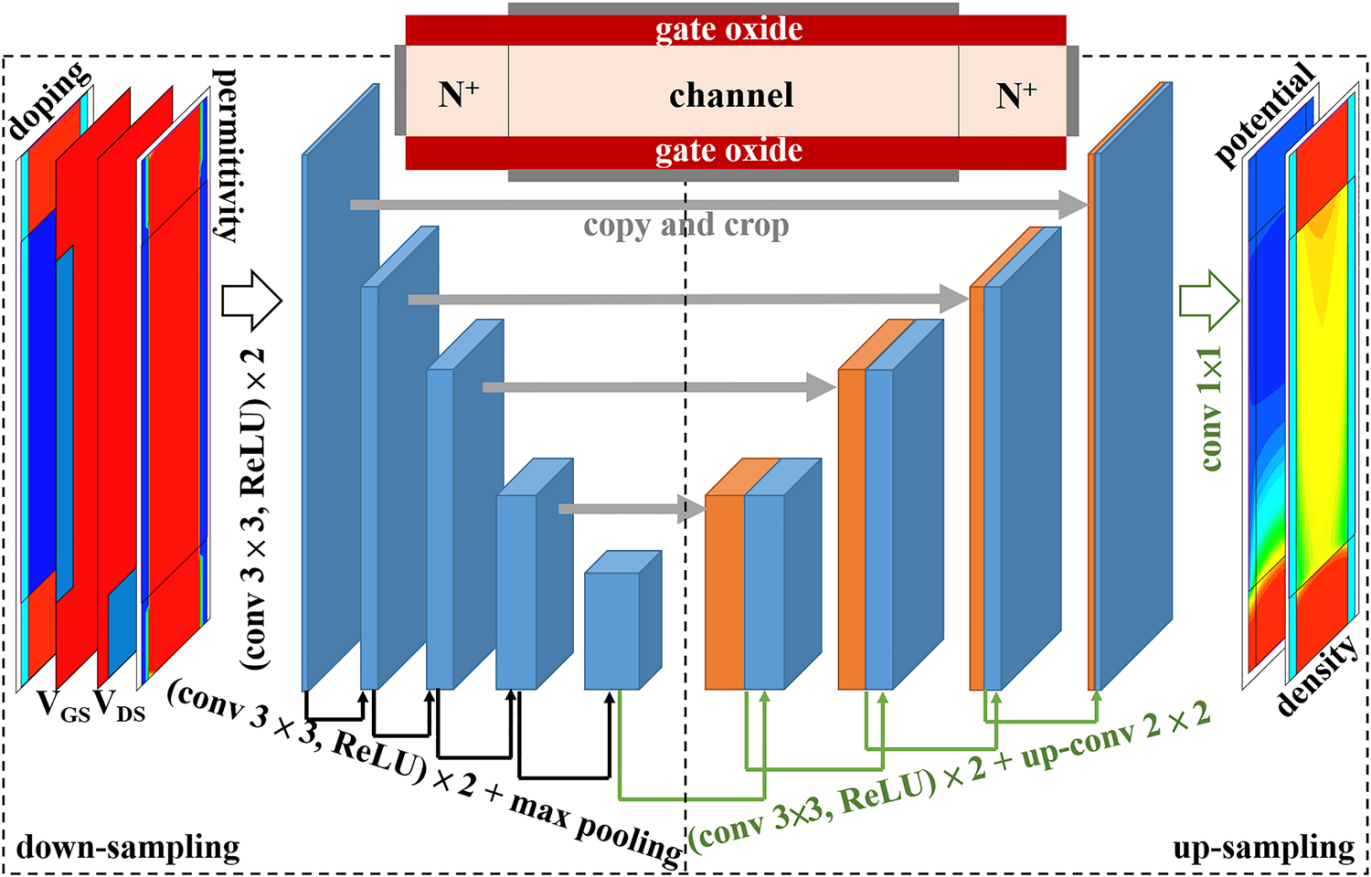
A schematic structure of a U-Net with down- and up-sampling procedures within the dashed frame. The down-sampling procedure begins with four input parameters matrixes, and then the data is processed by two convolutional layers and one max pooling layer. Each layer is copied and concatenated from the up-sampling to the down-sampling procedure. The upper insert is a schematic device structure with the following settings in TCAD simulations: doping density in source/drain (1 × 10^20^ cm^−3^) and channel (1 × 10^15^ cm^−3^) regions; channel thickness (5 nm) and length (30 nm); gate effective oxide thickness (*EOT* = 1 nm); gate work function (4.5 eV).

A 2D double-gate n-channel MOSFET is employed with all constant device parameters, defined in the caption of Fig. 1. Self-consistent calculation solving Poisson and drift–diffusion equations together with a quantum correction model is performed at drain bias *V*_*DS*_ = 0.6 V. By ramping gate bias *V*_*GS*_ = 0 ~ 0.6 V with a step of 0.01 V, a totally of 61 different sets of electrostatic potential and electron density distribution in a form of 2D matrix with a structured grid (0.2 nm in two directions) are extracted by linear interpolation from the simulator. With these data, we train two different U-Net models. Model 1 is trained by 7 sets of data at *V*_*GS*_ = 0 ~ 0.6 V with a step of 0.1 V and validated by another random 7 sets of data. For Model 2, we divide the 61 sets of data randomly so that 44, 12 and 5 of the results are used for training, validation and testing, respectively. The data category for the two models is summarized in Table 1. Compared to prior literatures^1–13^, thanks to the unique advantage of U-Net^17^, hundreds or even thousands of data are not needed to train the model.

**Table 1:** Data category and the mean and maximal (in brackets) absolute percentage errors (%) of testing data of the two U-Net models at *V*_*GS*_ = 0.29 V.

U-Net model	Model 1	Model 2
Training/validation/testing data (61 in total)	7/7/47	44/12/5
Electrostatic potential	0.86 (12.1)	0.14 (1.43)
Electron density	18.7 (725)	5.73 (71)

Figure 1 presents the schematic structure of the U-Net framework utilized in this work, which consists of down- and up-sampling procedures for data predictions. The down-sampling is a series of two convolutional layers and one max pooling layer with four input parameter matrixes. Our U-Net model is trained by feeding the two-dimensional input images, including relative permittivity, doping profile, *V*_*DS*_ = 0.6 V, and varying *V*_*GS*_. In the up-sampling, it replaces the max pooling with up-convolutional layer. The high-resolution features from down-sampling are combined with the up-sampling output, as indicated by the thin horizontal arrows. The output images are electrostatic potential and electron density distribution, respectively. This model only consists of fully convolutional layers, which is the so-called “fully convolutional network”^20^. The activation function of each convolutional layer is ReLU, except for the output layer of the model, which is due to the distribution of the physics parameters. All deep learning calculations are carried out with *Keras*. Backpropagation algorithm^21^ with gradient descent^22^ are employed for training.

## Results and discussions

**Figure 2:**
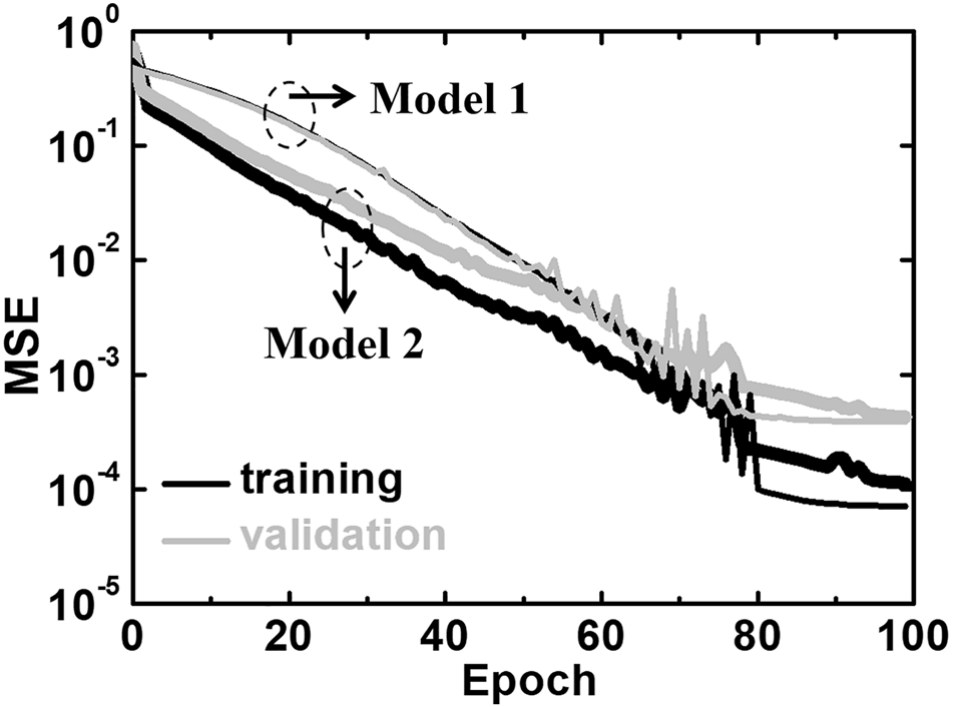
Training (black) and validation (grey) errors as functions of the learning epoch for Model 1(thin lines) and 2 (thick lines) trained by our U-Net. There are 200 steps in each epoch.

To compare with Model 1 and to enhance the robustness of the U-Net in this work, five-fold cross validation is used for Model 2, and a loss function in terms of the lowest mean-squared error (MSE) is shown in Fig. 2. Together with the fact that the number of training data is less than a hundred, the U-Net shows excellent learning efficiency (MSE < 10^−3^) within 80 epochs for both Model 1 and 2. Although Model 1 is trained by a limited data number, the mean percentage errors are fairly small as indicated in Table 1. However, compared to the mean percentage, the maximal percentage errors of 12.1 and 725% are higher for the electrostatic potential and electron density, respectively.

**Figure 3:**
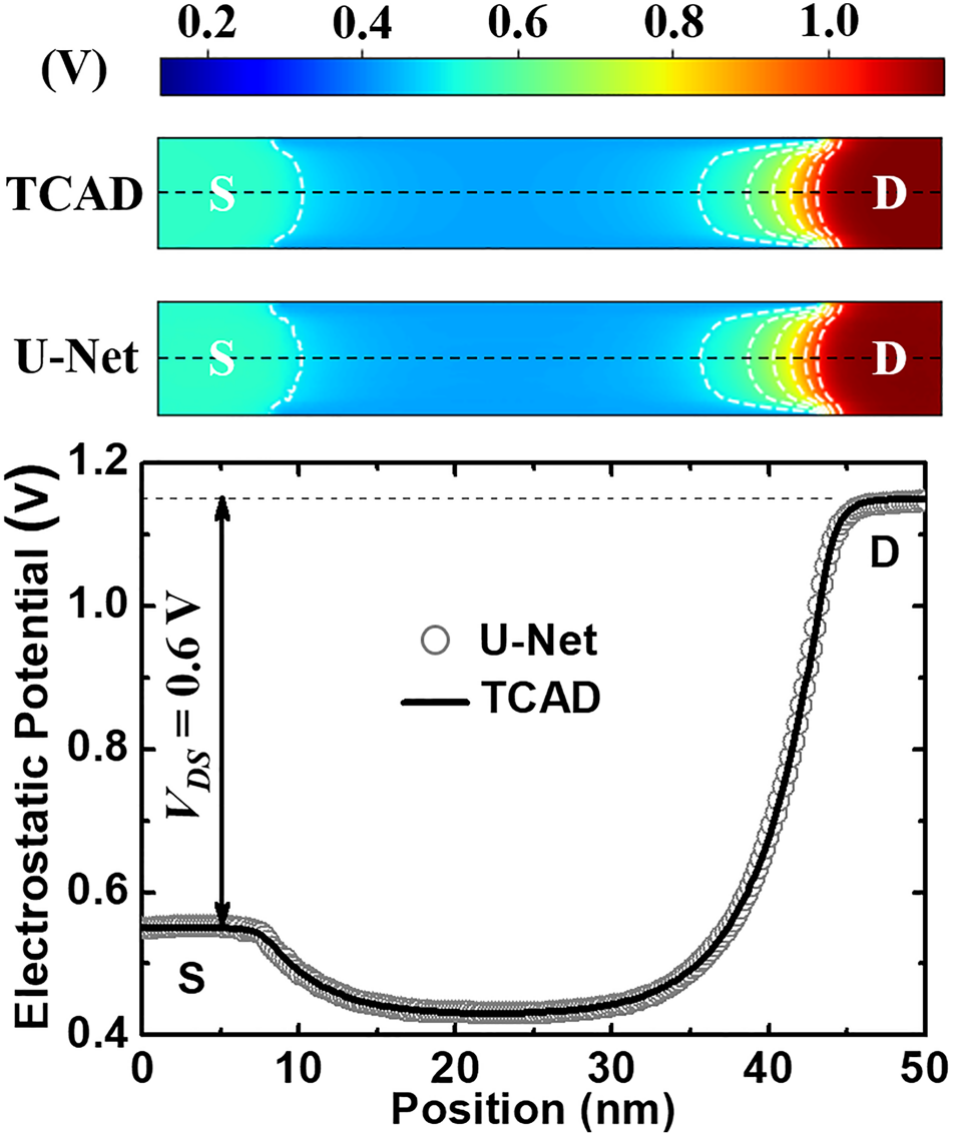
Comparisons between TCAD testing data and U-Net predicted results of electrostatic potential contour at *V*_*DS*_ = 0.6 V and *V*_*GS*_ = 0.29 V. The contour lines (white dashed) are plotted with a constant step of 0.1 V. One-dimensional (1D) profiles along the black dashed lines are showed at the bottom (*S* source, *D* drain).

**Figure 4:**
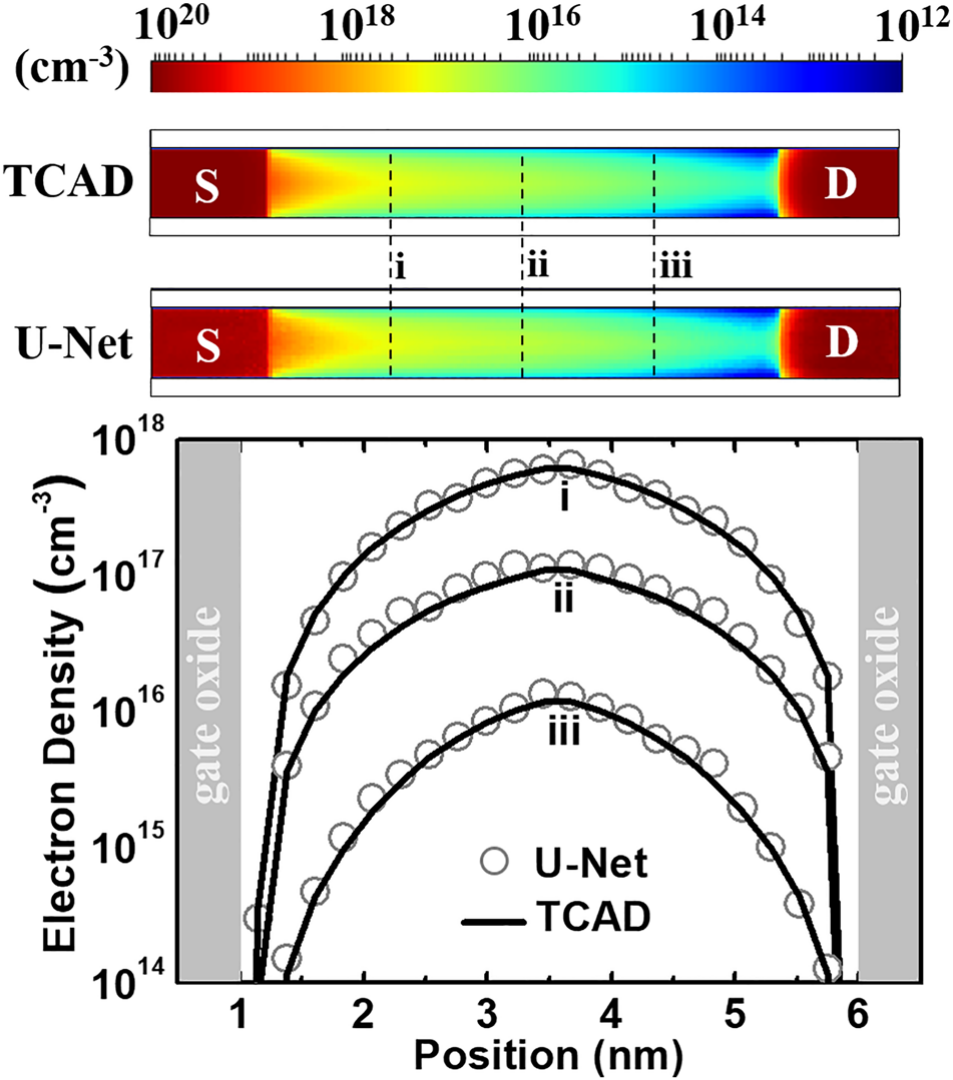
Comparisons between TCAD testing data and U-Net predicted results of electron density distribution at *V*_*DS*_ = 0.6 V and *V*_*GS*_ = 0.29 V. The white boxes represent oxide regions because there are no charges. 1D profiles along the black dashed lines are shown at the bottom.

Considering predictions by Model 2, Figs. 3 and 4 show the typical electrostatic potential contour and electron density distribution respectively at a given bias condition obtained by TCAD simulations, which is used as testing data. The potential and electron density profiles predicted by the U-Net match very well with the self-consistent TCAD results. Also, quantum-mechanically confined electron distribution can be literally captured by the U-Net model in Fig. 4.

**Figure 5:**
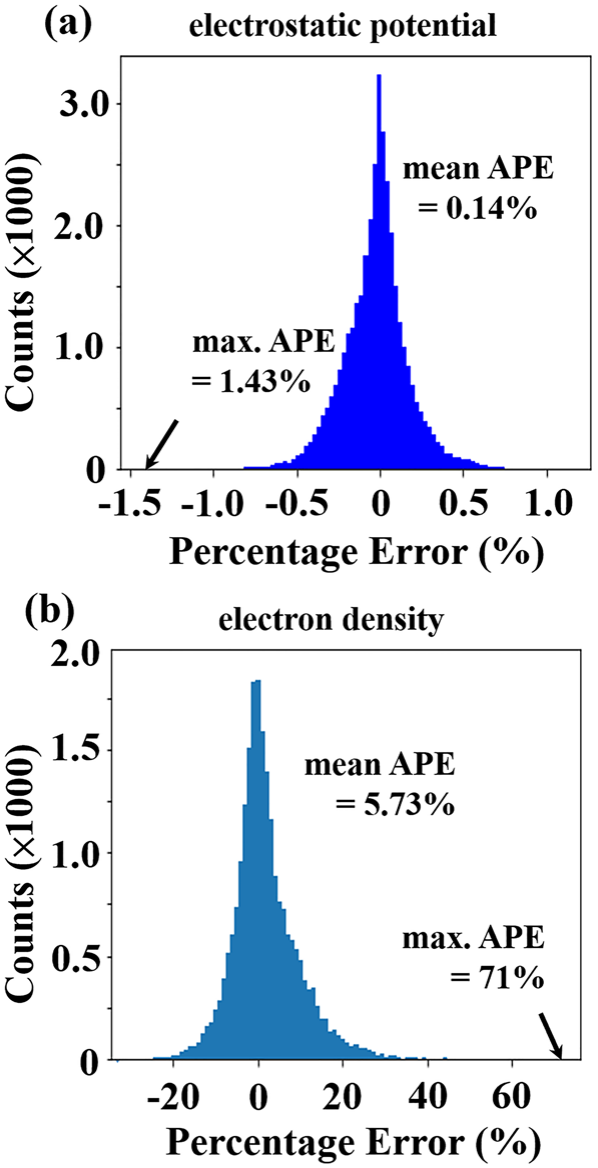
Percentage error histogram for the predicted electrostatic potential (**a**) and electron density (**b**). Each histogram compiles error values of 5 testing data and there are 7168 (4480) mesh points in each testing data of electrostatic potential (electron density), leading to 35,840 (22,400) total counts. Due to zero electron density in the gate oxide, the mesh points in the regions are uncounted, resulting in fewer total counts in (**b**). *APE* indicates absolute percentage error.

Up to now, with Model 2, only one of the five testing data is discussed in Figs. 3 and 4. To provide a full picture of the prediction accuracy of the trained U-Net, the errors for the five testing data are presented in Fig. 5. The mean and maximal values of the errors of electrostatic potential (electron density) are about 0.14% and 1.43% (5.73 and 71%), respectively. As the prediction error of the potential is ignorable, that of the density seems somewhat larger. Assuming a true value of 1 × 10^18^ cm^−3^, in the other words, the trained U-Net would return mean and maximal values of 1.0573 × 10^18^ and 1.71 × 10^18^ cm^−3^ for the electron density, respectively. Considering a fact that the electron density varies exponentially in the device from about 1 × 10^10^ to 1 × 10^20^ cm^−3^ (see Fig. 4), the prediction is still fairly accurate as a predicted value does not deviate by a factor greater than 1.71.

Compared to Model 1, in Table 1, the mean and maximal errors of Model 2 are reduced by a factor of about 6 (3) and 8 (10) for the predictions of electrostatic potential (electron density), respectively. This can be attributed to the completeness of training data, which is the key to accurate predictions by NN-based models. As potential and electron density varying slowly and exponentially in a device have been accurately predicted (see Figs. 3, 4, and 5), it implies that U-Net may make accurate predictions for other physical quantities in 2D landscapes with a sufficient amount of training data.

The whole device structure (Si, oxide, and electrodes) has been included in the loop of the learning process (Fig. 1). This is important so that the U-Net model is able to predict accurate landscapes across different material interfaces and at the boundaries. This has been shown in Figs. 3 and 4. It should be noted that as no charges are present in the gate insulator (no traps or gate leakage assumed in TCAD), the electron density vanishes in the oxide. However, this may cause a numerical problem when calculating the error on each mesh point in the oxide regions, which is explained in Fig. 5.

**Figure 6:**
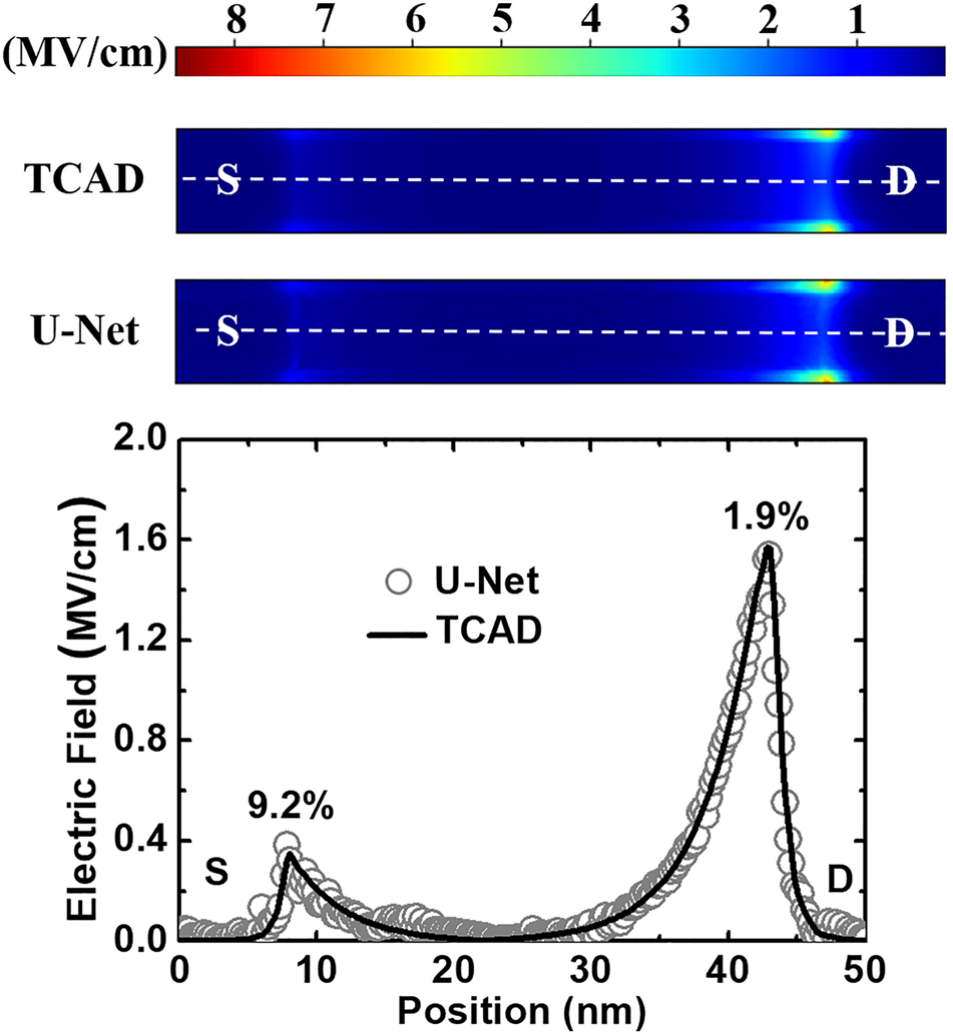
Comparisons between electric field extracted from TCAD simulations and deduced from the electrostatic potential contour predicted by U-Net at V_DS_ = 0.6 V and V_GS_ = 0.29 V. The 1D profile (bottom) is extracted from the 2D landscapes (upper) along the dashed lines. The errors of the maximum values at the source and drain junctions are about 9.2 and 1.9%, respectively.

Because our U-Net has not been trained with electric field data, it is not capable of making predictions for this physical quantity. However, the theoretically electric field can be determined by the gradient of the electrostatic potential predicted by the U-Net. Figure 6 shows the electric field extracted from the TCAD simulations and derived from the electrostatic potential of U-Net. Overall, U-Net result can duplicate the main features that can be observed in the TCAD landscape. Quantitatively, according to the profiles along the channel direction, the maximal error of the electrical field between TCAD and U-Net is smaller than 10%. On top of the error from U-Net prediction itself, the error may also originate from the value deviation between the unstructured (TCAD) and structured (U-Net) grids during training data generation. It is worth noting that the error is small in a region where the physical quantity varies more significantly, e.g. 1.9% at the drain junction. This is consistent with the observation of biomedical image analysis by U-Net.

**Figure 7:**
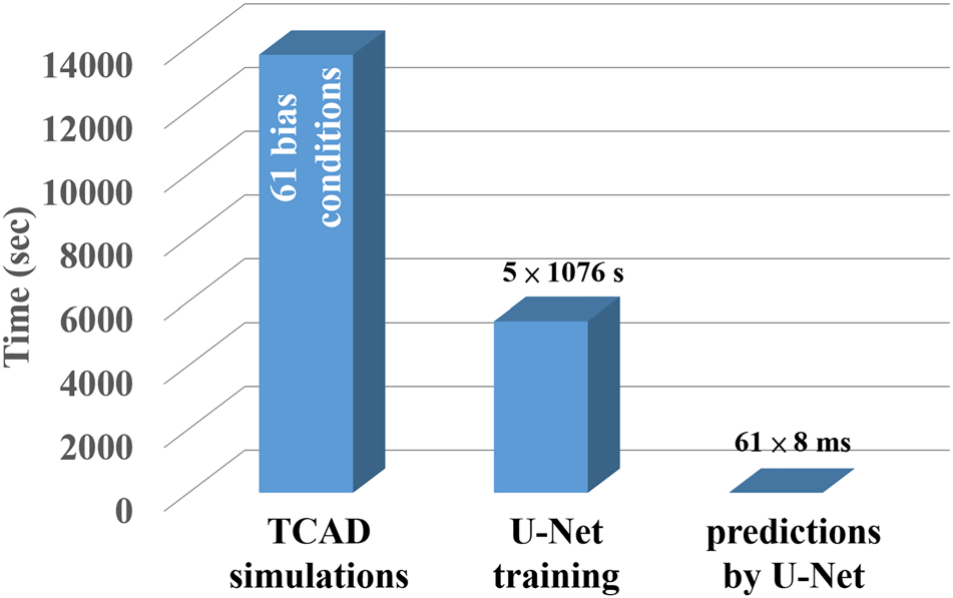
Run time of total simulations with TCAD, U-Net training with five-fold cross-validation and U-Net prediction for 61 sets of physical quantities (electrostatic potential and electron density distribution).

Run time of generating TCAD data with 61 bias conditions, training a U-Net model with five-fold cross-validations, and making predictions for a set of 2D landscapes of electrostatic potential and electron density distribution are benchmarked in Fig. 7. With the same computational resource, the calculation time of a trained U-Net is on averagely 2.8 × 10^4^ times faster than the traditional approach for one set of predictions. Therefore, this method may have the potential to deal with much more complicated cases with many other physical quantities.

**Figure 8:**
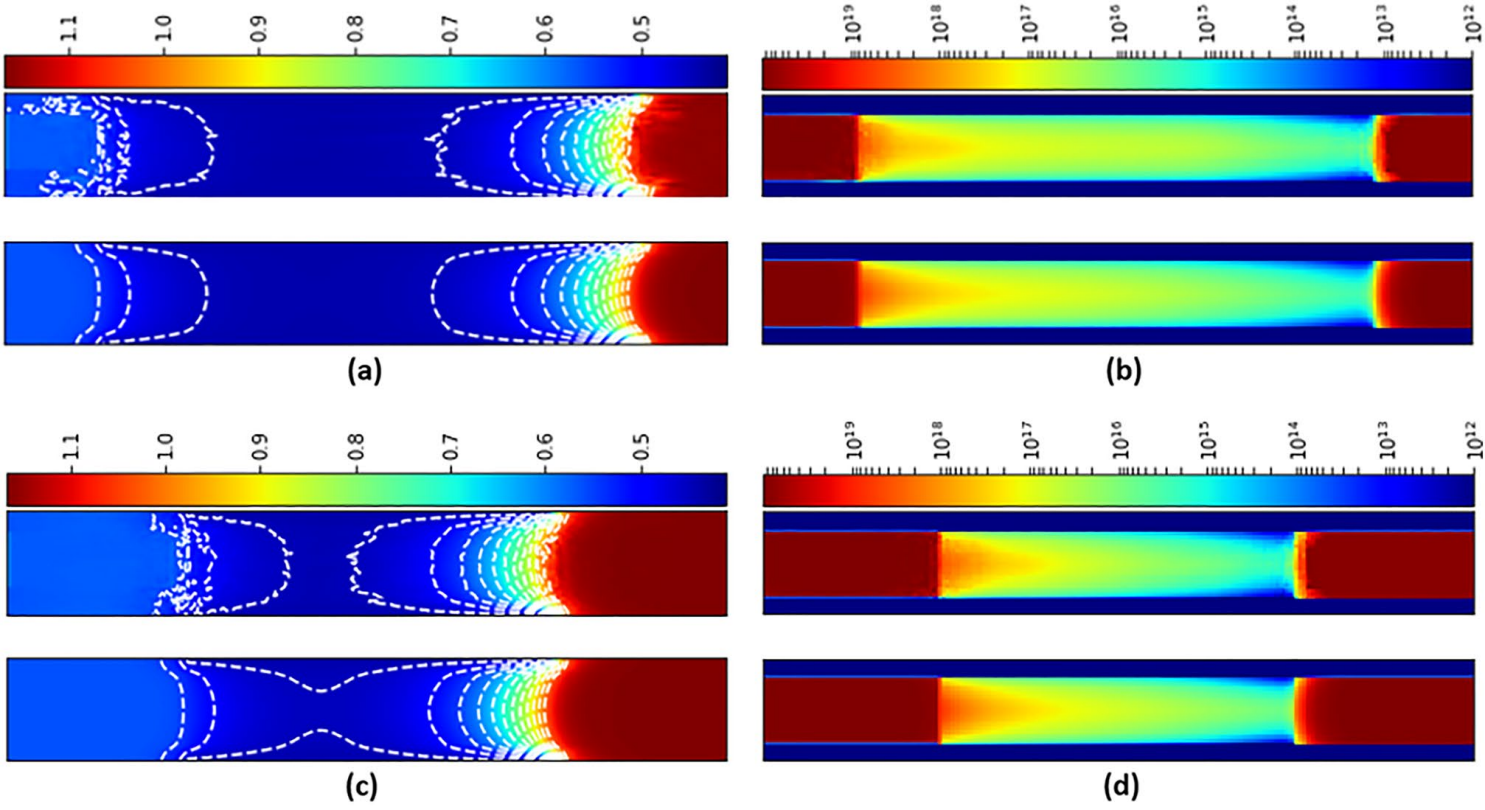
Comparisons of electrostatic potential and electron density between U-Net (upper) and TCAD (lower) for *L*_*ch*_ = 32 (**a,b**) and 22 (**c,d**) nm.

In order to evaluate the capability of the model for extrapolated predictions, we have trained another U-Net model based on training data with different channel length L_ch_ = 30, 28, 26 and 24 nm, named Model 3. Extrapolated predictions are made and images are shown below in Fig. 8 for L_ch_ = 32 and 22 nm. At a first glance, predictions by U-Net seem in good agreement with the TCAD results. Compared to interpolated prediction (Fig. 5), Table 2 shows the larger but acceptable mean and maximal errors of the extrapolated predictions of the models for the extrapolation cases of *L*_*ch*_ = 22 and 32 nm. For the electrostatic potential, it is worth to note that while the maximum errors are around 10.3% and 15.5%, the mean perdition error remains very low values of 0.79% and 0.73% for the cases of *L*_*ch*_ = 22 and 32 nm, respectively. It indicates that not only for ultimate predictions, but U-Net predictions can be very useful for a fast and appropriate initial solution of electrostatic potential for device simulations^14^. For the electron density, while the maximum deviation ratio of the predictions by Model 3 is about 4 and 6, the mean deviation ratio is only about 1.22 and 1.36. The mean error is actually neglectable. Taking a true doping density of 1 × 10^20^ (1 × 10^12^) cm^−3^ at source/drain (channel) for example, 1.36 × 10^20^ (1.36 × 10^12^) cm^−3^ will not result in noticeable difference on the device performance. We note that the bigger error of the maximum value is mainly located at the interfaces between channel and source/drain, where the electron density changes significantly from 1 × 10^12^ to 1 × 10^20^ cm^−3^. Compared to this enormous density gradient in a few nanometers at the interfaces, the local maximum error (deviation ratio of 4 ~ 6) at the interfaces is also ignorable in device simulations. The error at the interfaces originate from the low resolution of the linearly-spaced grid matrix of the image data, which is difficult to represent the exponential variation of the physical quantity (1 × 10^12^ ~ 1 × 10^20^ cm^−3^) in the extrapolated predictions. We also note that mesh size effect is a common and critical problem in mesh-based simulation theory. Therefore, mesh refinement procedure is usually adopted in the hot zone in numerical simulations, e.g., localized stress concentration of mechanism in structural mechanics analysis^23^ and interface between air and earth’s surface in geo-electromagnetic modelling^24^. However, the exponentially varying mesh refinement, which is not available, seems particularly crucial for the prediction error in our U-Net model for device simulations^25^.

**Table 2:** Mean and maximal (in brackets) percentage errors (%) of extrapolation cases (L_ch_ = 32 and 22 nm) of U-Net models trained by data with *L*_*ch*_ = 30, 28, 26 and 14 nm (Model 3) at VGS = 0.29 V.

U-Net model	Model 3	
	L_ch_ = 32	L_ch_ = 22
Electrostatic potential	0.79 (10.3)	0.73 (15.5)
Electron density	22.4 (398)	36.2 (632)

It is the first time to use U-Net to establish the prediction model for semiconductor devices. Our U-Net is trained by images with values on the matrix mimicking device structures, allowing extraction of the correlation between neighboring pixels through the convolution operation and output corresponding to the physical properties. The input and output are structurally and physically correlated as shown in Fig. 1. Hence, our model can be trained with a much less amount of data to efficiently and accurately predict the different physical quantities with low mean error. For example, although the maximum error of electrostatic potential for Model 1 trained with only 7 dataset is around 12.1% (mainly caused at the boundary), the mean error of 0.86% is still very low. This model is also useful to quickly predict appropriate initial solution of electrostatic potential for device simulation^25^. Furthermore, the model has the potential to be applied to rapidly screen high-performance devices and understanding the electrical properties of the device in a high-throughput manner, with quantum correction and without enormous computational cost of TCAD simulation.

Eventually, we point out that since the deep learning model is highly dependent on the behavior of training data, the model can only learn the behavior that they have seen. As the physical quantity is set as constant in training, it is not possible to be a variant quantity in perdition. Topology, size, doping density, and V_DS_ are the example parameters in this case. This work aims to show for the first time how to utilize U-Net and predict physical quantities considering quantum effects in a 2D device system. Therefore, to extend the capability of the model for further applications, a training dataset with more variable can be considered. The more consideration of variables used in the training dataset is, the better capability of the model will be. In addition, it is also capable to use the U-Net model in a 3D device system to greatly reduce the computing time.

## Conclusion

Towards explainable NN-based predictions for device simulations, apart from electrical curves, we also need physical quantities for better insights into device physics. By training a U-Net model with self-consistent TCAD data, the U-Net shows the high accuracy of predictions for physical quantities in two-dimensional landscapes, including electric field, electrostatic potential and quantum-mechanically confined electron density distribution. Our results show that the trained U-Net performs very well when predicting physical quantities that varies slowly and exponentially in a device. Hence, it is believed that U-Net can be trained to accurately predict other physical quantities of electron devices as well. This work has paved the way for interpretable predictions of device simulations based on convolutional neural networks.

## Data Availability

Data inquiries can be directed to the corresponding author.
